# Decoding the transcriptional heterogeneity, differentiation lineage, clinical significance in tissue-resident memory CD8 T cell of the small intestine by single-cell analysis

**DOI:** 10.1186/s12967-024-04978-2

**Published:** 2024-02-25

**Authors:** Jialing Fang, Jun Lei, Boxiao He, Yankang Wu, Peng Chen, Zaiqiao Sun, Ning Wu, Yafei Huang, Pengcheng Wei, Lei Yin, Yongshun Chen

**Affiliations:** 1 State Key Laboratory of Virology, Hubei Key Laboratory of Cell Homeostasis, College of Life Sciences, Department of Clinical Oncology, Renmin Hospital of Wuhan University, Wuhan University, Wuhan, China; 2 Department of Laboratory Medicine, Xixi Hospital of Hangzhou, Hangzhou, China; 3 Department of Immunology, School of Basic Medicine, Tongji Medical College, Huazhong University of Science and Technology, Wuhan, China; 4 Tongji Medical College, Huazhong University of Science and Technology, Wuhan, China; 5 School of Medicine, Guangxi University, Nanning, 530004 China; 6 Department of Immunology and Genomic Medicine, National Jewish Health, Denver, CO 80206 USA

**Keywords:** Sc-RNA-seq, Tissue resident memory T cell, Intestine, T cell differentiation, Clinical cancer significance

## Abstract

Resident memory T (Trm) cells which are specifically located in non-lymphoid tissues showed distinct phenotypes and functions compared to circulating memory T cells and were vital for the initiation of robust immune response within tissues. However, the heterogeneity in the transcriptional features, development pathways, and cancer response of Trm cells in the small intestine was not demonstrated. Here, we integrated scRNA-seq and scTCR-seq data pan-tissue T cells to explore the heterogeneity of Trm cells and their development pathways. Trm were enriched in tissue-specific immune response and those in the DUO specially interacted with B cells via TNF and MHC-I signatures. T cell lineage analyses demonstrated that Trm might be derived from the T_CD4/CD8 subset within the same organ or migrated from spleen and mesenteric lymph nodes. We compared the immune repertoire of Trm among organs and implied that clonotypes in both DUO and ILE were less expanded and hydrophilic TRB CDR3s were enriched in the DUO. We further demonstrated that Trm in the intestine infiltrated the colorectal cancer and several effector molecules were highly expressed. Finally, the TCGA dataset of colorectal cancer implied that the infiltration of Trm from the DUO and the ILE was beneficial for overall survival and the response to immune checkpoint blockade.

**Supplementary Information:**

The online version contains supplementary material available at 10.1186/s12967-024-04978-2.

## Highlights

1. The result uncovers the heterogeneity of tissue-resident memory T cell subsets of distinct intestine organs.
2. The result uncovers the heterogeneity of the differentiation pathways of tissue-resident memory T cell subsets of distinct intestine organs which might be derived from the T_CD4/CD8 subset within the same organ or migrated from distinct organs.
3. T cells from jejunum lamina propria (JEJLP) and jejunum epithelium (JEJEPI) showed overlapped clonotypes which were more clonally expanded.
4. Tissue-resident memory T cell subsets of DUO and ILE in cancer showed acute immune response as well as apoptotic processes.
5. The infiltration of tissue-resident memory T cell subsets of DUO and Ileum (ILE) benefited the overall survival of the colorectal cancer patient.
6. The infiltration of tissue-resident memory T cell subsets of DUO and Ileum (ILE) benefited the response of the immune checkpoint blockade therapy of the colorectal cancer patient.

**Supplementary Information:**

The online version contains supplementary material available at 10.1186/s12967-024-04978-2.

## Introduction

Human memory CD8 T cells are diverse in molecular phenotypes and biological functions and can be divided into three subsets including those that circulate in the peripheral blood, those that are located in lymphoid tissues, and those that are mainly resident in non-lymphoid tissues [20]. Circulating memory T cells include effector memory T cells that migrate in the red pulp of the spleen and infected non-lymphoid tissues [19] and central memory T cells that express several chemokine receptors and lymphoid homing receptors [58]. Resident memory T cells, referred to as Trm cells are identified in non-lymphoid tissues and different from circulating memory CD8 T cells [28, 29]. Trm cells don’t express lymphoid homing receptors but express molecules preventing tissue egress like CD69 [27] and integrins like CD103 and CD49a that guarantee their tissue residency [6, 29]. Previous studies demonstrated that Trm persist for years and markers of Trm were reduced in infancy which indicated the full maturity of Trm might require extrinsic factors confined to adult tissue microenvironment [46].

The intestine is enriched in tissue-resident lymphocytes which are mainly resident in lamina propria (LP) and epithelium (EPI) [48], constituting a barrier against pathogen entry. Therefore, intestinal Trm cells play a role in immune homeostasis and immune protection. During the intestine inflammation, TGF-β is important in the development and maintenance of CD8 Trm cells in the intestine [8, 14], and upregulation of integrin and chemokine receptor CCR9 are necessary in the homing to the intestine mucosa of CD8 Trm cells [56].

Trm cell expression CD103 was reported to exist in multiple human solid cancers including high-grade serous ovarian cancer (HGSOC) [52], endometrial adenocarcinoma [54], NSCLC [35], mesothelioma [22], and melanoma [5] and infiltrating T cell populations showed Trm-like phenotypes like the expression of CD69 and several inhibitory checkpoint molecules. Furthermore, previous studies reported that Trm cell infiltration was strongly correlated with survival and dominated the protective response in HGSOC [52] and urothelial cancer [49], breast cancer [51], endometrial cancer [54], and lung cancer [10]. A Trm gene signature acquired from scRNA-seq datasets of TNBC patients was predicted for their survival and could be utilized for the prediction of response to ICB therapy [40].

To dissect the transcriptional features, differentiation pathways, immune repertoire, and immune response of small intestine resident memory T cells which has not been fully demonstrated, we analyzed scRNA-seq and scTCR-seq datasets of T cells of distinct organs collected from a recently published study [11]. We showed that the Trm_gut_CD8 subset was specifically located in distinct regions of the intestine. We further revealed the heterogeneity in transcriptomic features and enriched signaling pathways of Trm_gut_CD8 in distinct organs. Trm_gut_CD8 in both DUO and ILE might be related to immune effector processes. The differentiation of the Trm_gut_CD8 in the DUO might be mediated by the interaction with B cells via TNF, LCK, CD48, and MHC-I pathways. It was also revealed that the Trm_gut_CD8 might be derived from T_CD4/CD8 within the same organ or migrated from other organs including SPL and MLN. We compared the immune repertoire of the Trm_gut_CD8 from distinct organs and implied that Trm_gut_CD8 clonotypes were more expanded in the jejunum (JEJ). At last, we revealed the infiltration of Trm_gut_CD8 in colorectal cancer and demonstrated that the infiltration of Trm_gut_CD8 specifically derived from DUO and ILE benefitted the overall survival and response to immune checkpoint blockade therapy which might be utilized as prognosis markers.

## Results

### The single-cell profiling of T cells among human organs

**Fig. 1:**
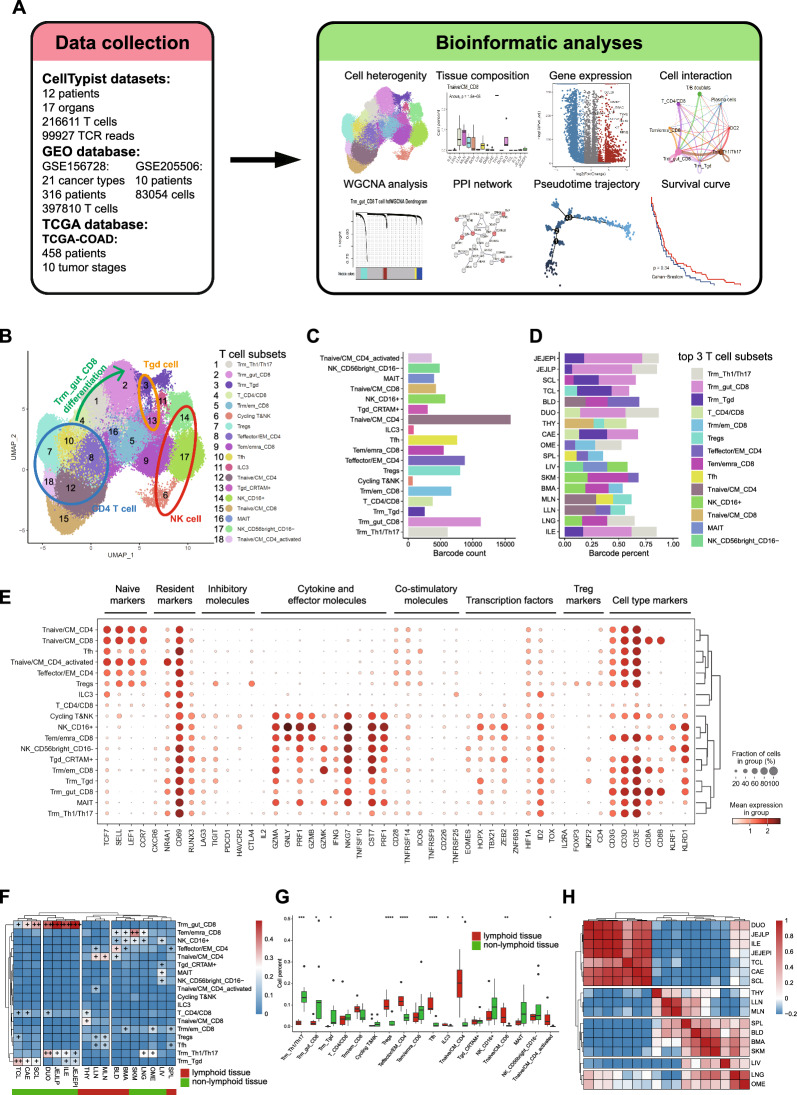
The single-cell transcriptomic profiling of T cell subsets among human organs. **A** Schematic diagram of the experimental workflow. **B** UMAP plot showing single cells collected from the CellTypist database, labeled by T cell subsets. **C** The bar plot indicates the overall barcode count of distinct T cell subsets. **D** The bar plot indicates the distribution of the top3 T cell subsets in each organ. **E** The dot plot indicates the expression of several marker genes in T cell subsets. **F** The heatmap indicating the distribution of T cell subsets in each organ. Cells with a proportion greater than 0.5 are labeled as “ +  +  + ”. Cells with a proportion greater than 0.3 are labeled as “ +  + ”. Cells with a proportion greater than 0.1 are labeled as “ + ”. **G** The box plot indicating the frequency of T cell subsets in lymphoid tissues and non-lymphoid tissues. **H** The heatmap indicates the correlation and clustering of the cell composition of each organ

To profile the heterogeneity of T cell composition among distinct human organs, we collected the scRNA-seq data of distinct T cell subsets from distinct human organs derived from a recently published study [11]. This dataset contained 216611 T cell barcodes from 17 organs of 12 patients (Fig. 1A). After quality control, high-quality barcodes whose mitochondrial gene percent is under 5% were eliminated. Barcodes from distinct cell subsets, donors, and organs showed similar sequencing quality (Additional file 1: Figure S1A–C). We identified the most variable genes (Additional file 1: Figure S1D) and those that yield the highest fraction of counts in each single cell (Additional file 1: Figure S1E). Samples from distinct organs and patients showed nearly no batch effects after the integration (Additional file 1: Figure S1F).

According to the published data, 18 T cell subsets were identified and visualized with the UMAP embedding graph (Fig. 1B). T_CD4/CD8, Trm_Th1/Th17, and Trm_gut_CD8 are located linearly which might suggest the differentiated pathway from T_CD4/CD8 to tissue-resident T cells. CD4 T cells subsets were located close to each other which was similar to NK cells and Tgd cells. Distinct samples had diverse barcode sizes (Fig. 1C and Additional file 1: Figure S2A). The Tnaive/CM_CD8 subset was mainly located in the THY (thymus) while the Tnaive/CM_CD4 subset was located in the BLD (blood) and lymph nodes. The T_CD4/CD8 subset was not only located in the THY but also was detected in the DUO (duodenum) and the CAE (caecum) which might be resulted from the migration of the T_CD4/CD8 among organs. The Trm_gut_CD8 subset was restrictedly located in distinct regions of the intestine which implied a specific differential phenotype of the Trm subset. Effector T cell subsets were preferred to be located in BLD, lymph nodes, BMA (bone marrow), and SPL (spleen) (Fig. 1D and Additional file 1: Figure S2B).

T cell subsets were heterogenous in the expression of several marker genes (Fig. 1E). Tnaive/CM_CD4 cells were transcriptionally close to Tnaive/CM_CD8 cells. The Treg subset showed high levels of inhibitory molecules. Several NK cell subsets and T cell subsets showed high levels of cytokines and effector molecules which might imply their functions in the immune response. CD69 and a lack of surface proteins including CCR7, CD62L, and CX3CR1 were utilized to identify CD8 Trm cells under steady condition [44]. Trm cells also expressed several inhibitory molecules which might limit inflammation and prevent tissue injury [50]. We demonstrated the overall distribution of T cell subsets in distinct organs (Fig. 1F). The Trm_gut_CD8 subset was mainly located in four intestine tissues including DUO, ILE (ileum), JEJEPI (jejunum epithelial), and JEJLP (jejunum lamina propria). We further noticed that cell subsets including NK_CD56bright_CD16, MAIT, Tgd_CRTAM + , Tem/emra_CD8, and NK_CD16 + which highly expressed cytokines and effector molecules were enriched in LIV (liver), SKM (skeletal muscle), and BMA implying that these organs might be related with the initiation of the response of innate immune and adaptive immune (Fig. 1E, F). It was implied that several tissue-resident T cell subsets were enriched in non-lymphoid tissues while Treg, Tfh, Teffector/EM_CD4, and Tnaive/CM T cell subsets were enriched in lymphoid tissues (Fig. 1G). The similarity of cell subsets among organs implied that the LIV showed a unique pattern of cell composition (Fig. 1H). Several intestine organs were clustered and organs including SPL, BLD, SKM, and BMA showed similar cell composition. We also implied that Trm_gut_CD8, T_CD4/CD8, and Trm_Tgd showed similar organ distribution in non-lymphoid tissues which is opposite to lymphoid tissues (Additional file 1: Figure S2C). T cells were maintained as naïve subsets in lymphoid tissues and the T cell activation marker CD69 was constitutively expressed in almost all tissues except the THY (Additional file 1: Figure S3A). T cells in lymphoid tissues showed higher expression of cytokines and effector molecules.

To illustrate transcriptional features of distinct T cell subsets, we calculated different genes (Additional file 1: Figure S3B) and implied that Trm_Tgd, Trm_Th1/Th17, and Trm_gut_CD8 as well as several naïve T cell subsets showed similar up-regulated genes and down-regulated genes which might be mediated by similar tissue microenvironments (Additional file 1: Figure S3C). We specifically illustrated the differential genes of the Trm_gut_CD8 subset and implied that this T cell subset was enriched in normal digestive system processes and immune response processes including mucosal immune response, inflammatory response, interferon, and cytokine production (Additional file 1: Figure S3D-E).

### Distinct molecular features of CD8 resident memory T cells among distinct gut regions

**Fig. 2:**
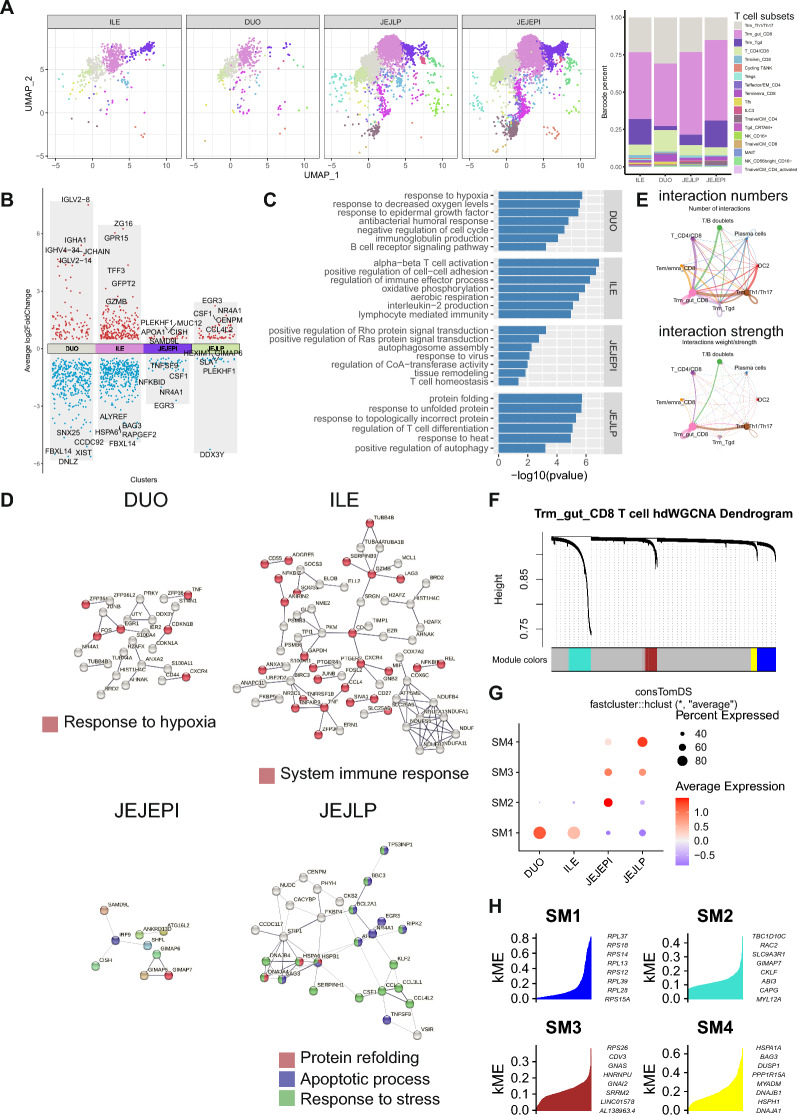
Single-cell transcriptome resolves heterogeneity of Trm_gut_CD8 in distinct intestine organs. **A** UMAP plot showing single cells in distinct intestine organs, labeled by T cell subsets. **B** The point plot indicating differentiation genes of Trm_gut_CD8 in distinct intestine organs. Genes with log2Foldchange greater than 0.5 and p value adjust less than 0.001 were annotated as differential genes. **C** The bar plot indicates enriched signaling pathways of Trm_gut_CD8 in distinct intestine organs. **D** Network showing the PPI in Trm_gut_CD8 in distinct intestine organs identified by STRING. **E** Circle plots indicating the number of interactions and the interaction strength of cell types in the DUO. **F** The dendrogram plot of the co-expression network of Trm_gut_CD8 in distinct intestine organs. **G** The dot plot indicating the expression features of gene modules in Trm_gut_CD8 of distinct intestine organs. **H** Genes in each module are ranked by kME using the PlotKMEs function

Trm cells can be found in almost all organs but are particularly enriched in front-line tissues including skin, lung, and intestine epithelium in humans [15, 32, 42]. It was assumed that Trm cells have developed distinct mechanisms to adapt to distinct microenvironments [3, 4, 30]. We noticed that the Trm_gut_CD8 subset was specifically enriched in several intestine organs including DUO, ILE, JEJEPI, and JEJLP, and therefore analyzed their heterogeneity in the distribution and gene expression of Trm_gut_CD8 that mediated their organ adaption (Fig. 2A). We illustrated differential genes (Fig. 2B and Additional file 1: Figure S4A) and enriched signaling pathways (Fig. 2C) of the Trm_gut_CD8 among four organs. We noticed that several genes related to BCR were up-regulated in the DUO and suggested that B cells might interacted with the Trm_gut_CD8 in the DUO. It was implied that the Trm_gut_CD8 in the DUO and the ILE showed similar differential genes (Additional file 1: Figure S4B). The Trm_gut_CD8 was enriched in response to decreased oxygen levels, B cell-related immunoglobulin production, and BCR signaling in the DUO while enriched in the immune effector process and aerobic respiration in the ILE (Fig. 2C). To extract specific gene signatures that represent the transcriptomic feature of each cluster, we took differential genes and their PPI interactions via the STRING tool [45]. The DUO-specific gene module was related to the response to hypoxia, and the ILE-specific gene module was related to the system immune response which suggested their role in the immune response (Fig. 2D and Additional file 1: Figure S4C).

Genes encoding the immunoglobulin were enriched in the Trm_gut_CD8 of the DUO and therefore we analyzed the receptor & ligand interaction network [21] among cell types in the DUO (Additional file 1: Figure S5A, B) and implied that T/B doublets and plasma cells interacted with the Trm_gut_CD8 (Fig. 2E). We found out that CD48, TNF, LCK, and MHC-I signaling pathways were significantly enriched between the Trm_gut_CD8 and the T/B doublets. We further identified specific receptor & ligand patterns that mediated the interaction between two clusters (Additional file 1: Figure S5C-E) and demonstrated that interactions that contributed significantly to the MHC-I signature were detected in the Trm_gut_CD8 and the T/B doublet (Additional file 1: Figure S5F).

We analyzed co-expression networks in the scRNA-seq data of the Trm_gut_CD8 in four organs using hdWGCNA [34] which is a comprehensive framework for the analyses of network inference, gene module identification, gene enrichment, and data visualization. After setting up the Seurat object for WGCNA which constructed the group information and the expression matrix, we included a function TestSoftPowers to perform a parameter sweep for different soft power thresholds and chose a soft power threshold as 4 for constructing the co-expression network (Additional file 1: Figure S6A). 4 significant gene modules were identified after the construction of the co-expression network (Fig. 2F). The score of each module and their expression among organs implied that SM1 was activated in both DUO and ILE, SM2 was specifically activated in the JEJEPI, SM4 was specifically activated in the JEJLP, and SM3 were activated in both JEJEPI and JEJLP (Fig. 2G). Besides, SM1 was negatively correlated with other modules while SM1 to SM3 were positively correlated (Additional file 1: Figure S6B). Next, we visualized hub genes determined by the eigengene-based connectivity (kME) and thus were highly connected within each module (Fig. 2H and Additional file 1: Figure S6C). Following gene ontology analyses implied that SM1 was enriched in ribosome activity as well as immune cell activation and differentiation. SM2 was enriched in immune response mediated by T cell receptor and antigen interaction. SM4 was enriched in chaperone-mediated protein folding and refolding upon the heat shock (Additional file 1: Figure S6D).

### Distinct differentiation pathways of CD8 resident memory T cells among distinct gut regions

Intestinal CD8 Trm cells are exceptionally heterogeneous and differentiate via diverse development pathways in mice and humans [14, 23, 33]. In our study, several tissue-resident T cell subsets like Trm_gut_CD8 were not restrictedly located in a specific organ but also appeared in distinct organs. Thus, we analyzed the T cell lineage to imply the differentiation pathway of distinct subsets using the STARTRAC [59] package based on the scRNA-seq data and paired scTCR-seq data. Distinct T cell subsets with the same TCR clonotype were assumed to have the same precursor T cell subset. The STARTRAC-expa index indicating the clonal expansion of TCR clonotype implied that T cell subsets including Trm_gut_CD8, Trm_gut, Trm/em_CD8, Tem/emra_CD8, Tnaive/CM_CD4, and MAIT showed high expansion index in both lymphoid and non-lymphoid tissues (Additional file 1: Figure S7A left). The STARTRAC-migr index indicating the migration of T cells among organs implied that T cell subsets except Treg, Tfh, and Tnaive/CM showed migration ability. Trm_gut_CD8, Trm_Tgd, and T_CD4/CD8 showed higher migration ability in non-lymphoid tissues compared to lymphoid tissues (Additional file 1: Figure S7A middle). The STARTRAC-tran index indicating the transition of distinct T cell phenotypes implied that T_CD4/CD8 showed higher transition ability in non-lymphoid tissues compared to lymphoid tissues. The STARTRAC-gini index indicating the diversity in clonotypes within the same T cell phenotype implied that Trm_gut_CD8 showed a high gini index which might suggest that it was derived from distinct precursor T cells (Additional file 1: Figure S7A right).

**Fig. 3:**
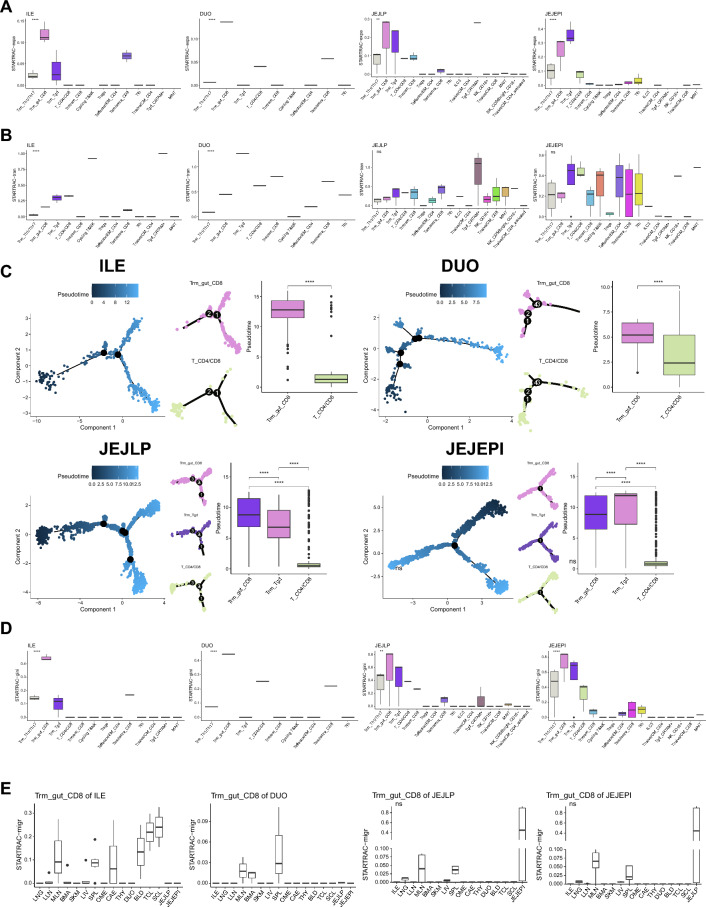
Trm_gut_CD8 lineage tracking in distinct intestine organs. **A** Boxplot indicating the STARTRAC-expa index of T cell subsets in distinct intestine organs. **B** Boxplot indicating the STARTRAC-tran index of T cell subsets in distinct intestine organs. **C** Pseudo-time trajectory analyses of the differentiation route of the Trm_gut_CD8 subsets in distinct intestine organs. **D** Boxplot indicating the STARTRAC-gini index of T cell subsets in distinct intestine organs. **E** Boxplot indicating the STARTRAC-migr index of Trm_gut_CD8 subsets in distinct intestine organs

To further analyze the differentiation pathway of Trm_gut_CD8 subsets in organs including DUO, ILE, JEJEPI, and JEJLP, we performed the STARTRAC pipeline on the selected scRNA-seq data and scTCR-seq data. The Trm_gut_CD8 was significantly expanded in the above four organs (Fig. 3A and Additional file 1: Figure S7B). The Trm_gut_CD8 showed a medium STARSTRAC-tran index which implied that partial Trm_gut_CD8 were derived from other T cell subsets within the same organ (Fig. 3B). The comparison of TCR clonotypes of subsets within the same organ suggested that the Trm_gut_CD8 might be derived from the T_CD4/CD8 subset (Additional file 1: Figure S7C). Besides, the Trm_gut_CD8, Trm_Tgd, and Tem/emra_CD8 showed overlapping clonotypes in JEJLP and JEJEPI (Additional file 1: Figure S7C bottom). The pseudo-time trajectory analyses of distinct T cell subsets within the same organ also suggested that the Trm_gut_CD8 might be derived from the T_CD4/CD8 (Fig. 3C). The Trm_gut_CD8 of four organs showed high levels of STARTRAC-gini index which suggested its diversity in lineage (Fig. 3D). Furthermore, we tried to find out the migration pathway of the Trm_gut_CD8 among organs. The Trm_gut_CD8 of four organs showed similar TCR clonotypes to those derived from SPL and MLN (Fig. 3E). Besides, partial Trm_gut_CD8 clonotypes in the ILE were specifically similar to those clonotypes derived from organs including CAE, BLD, TCL, and SCL. Minor Trm_gut_CD8 clonotypes in the DUO were similar to those derived from BMA. BMA contains T cells that react to multiple acute and persistent viruses and are not similarly present in the BLD [36, 47]. BMA memory CD8 T cells exhibit enhanced effector function [39, 60] and therefore Trm_gut_CD8 in the DUO which derived from the BMA showed an evident immune response in the cancer sample (Fig. 5D). The majority of the clonotypes of the Trm_gut_CD8 in the JEJLP also appeared in the JEJEPI (Additional file 1: Figure S7D). Finally, we systematically compared clonotypes of the Trm_gut_CD8 in the selected organ with other T cell subsets of other organs to illustrate possible differentiation pathways (Additional file 1: Figure S7E).

### The single-cell immune repertoire profiling of CD8 resident memory T cells among distinct gut regions

**Fig. 4:**
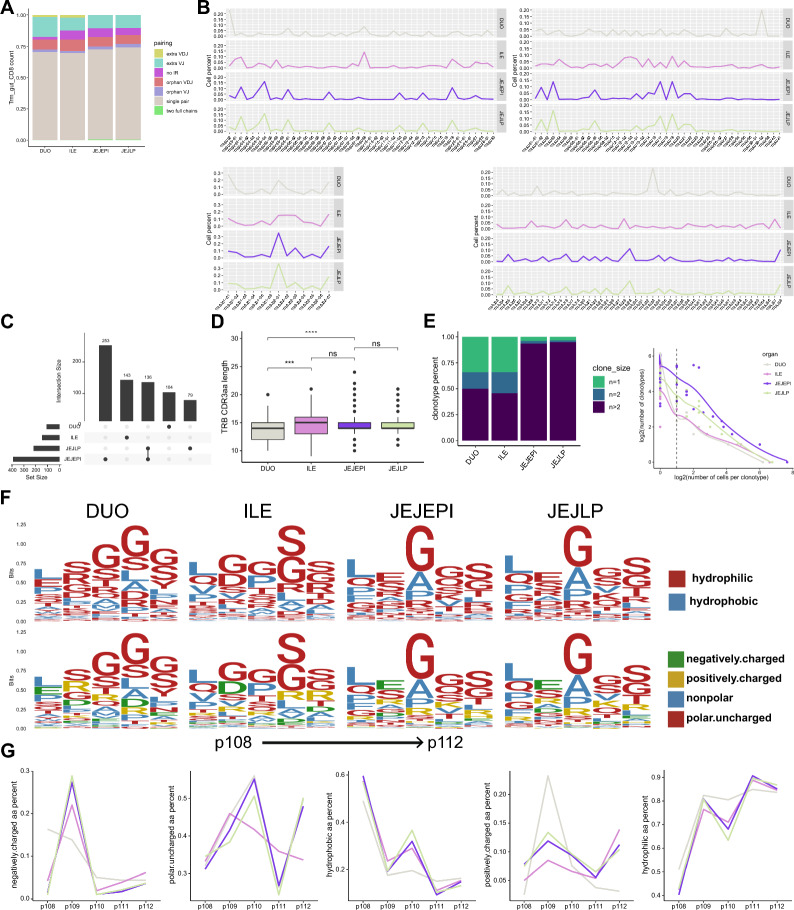
Immune repertoire analyses of clonotypes of Trm_gut_CD8 from distinct intestine organs. **A** The bar plot indicates the percent of barcodes detecting TCR counts. **B** Line plots indicate the proportion of TCR VJ gene usage. **C** The bar plot indicates the overlapping of TRB CDR3 among distinct intestine organs. **D** The box plot indicating the length of TRB CDR3aa among distinct intestine organs. **E** The bar plot and the point plot indicate the percent of clonotypes of distinct expansion degrees among organs. **F** Ggseqlogo of the composition of amino acids within the CDR3 middle region among distinct organs. **G** Line plots indicating the composition of amino acids in distinct positions among organs

**Fig. 5:**
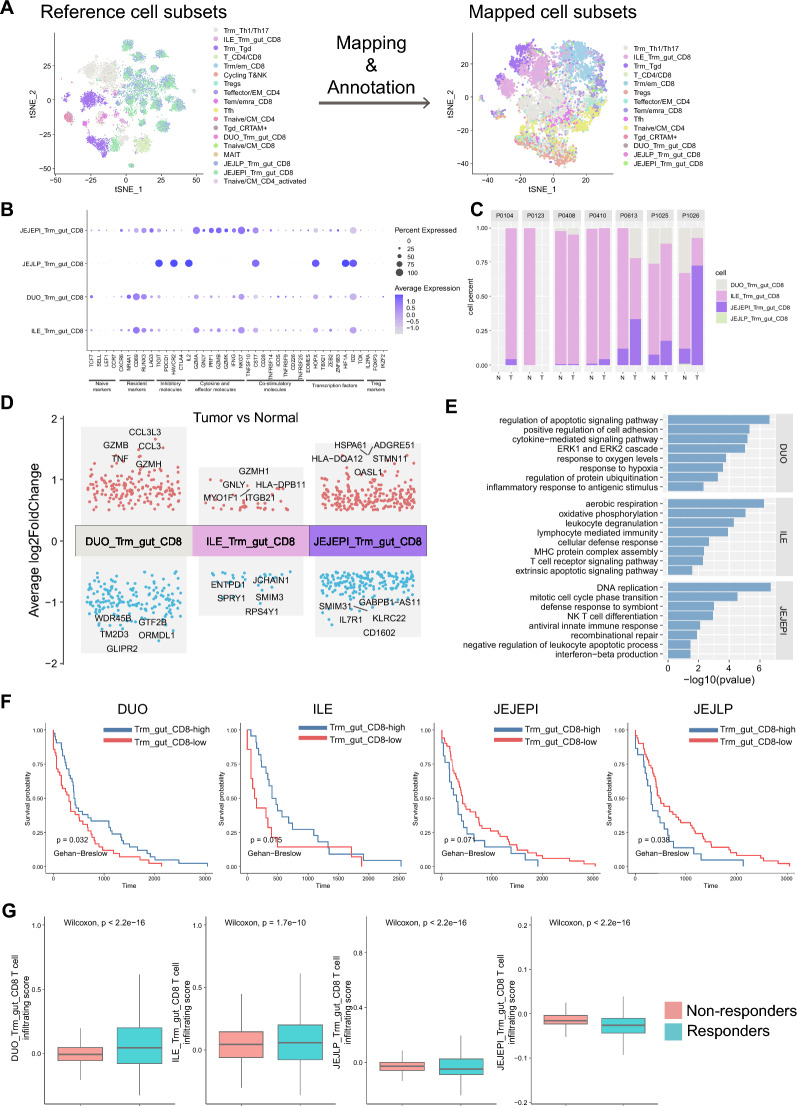
The function of Trm_gut_CD8 from distinct intestine organs in colorectal cancer and its clinical significance. **A** tSNE plots indicating the mapping and annotation from reference dataset to mapped scRNA-seq dataset including T cells of paired tumor and normal samples. **B** The dot plot indicating the expression levels of marker genes among predicted T cell subsets derived from distinct organs. **C** The bar plot indicates the proportion of predicted T cell subset between paired tumor and normal samples. **D** The differential genes of tumor Trm_gut_CD8 subsets of distinct organs compared to the normal sample. Genes with log2Foldchange greater than 0.5 and p value adjust less than 0.001 were annotated as differential genes. **E** The bar plot indicating the enriched signature of genes mentioned in (**d**). **F** Overall survival curves of colorectal cancer patients stratified by the infiltration scores of Tmr_gut_CD8 derived from distinct intestine organs. **G** Box plots indicating the correlation between the infiltration of Trm_gut_CD8 cells of distinct organs and the response to the immune checkpoint blockade therapy in the colorectal scRNA-seq data

To illustrate the differentiation pathway of the Trm_gut_CD8 from the immune repertoire perspective, we compared the immune repertoire of the Trm_gut_CD8 in the above four organs. More than 95% of the Trm_gut_CD8 detected corresponding TCR information (Fig. 4A). The usage of TCR VJ genes implied that T cells in the JEJEPI and JEJLP had similar VJ gene patterns while TCRs in the DUO were restricted in several specific VJ genes (Fig. 4B). There was partial overlapping of TRB clonotypes only between the JEJEPI and the JEJLP (Fig. 4C). TRB clonotypes in the DUO showed short CDR3 amino acid length compared to other organs (Fig. 4D). It was implied that TRB clonotypes of the Trm_gut_CD8 in the JEJEPI and the JEJLP were almost expanded (Fig. 5E). Furthermore, we tried to illustrate the difference in the amino acid composition in each specific position of TRB clonotypes among organs using TiRP package [24] (Fig. 4F) which implied that hydrophilic amino acids were more enriched in the DUO while negatively charged and hydrophobic amino acids were more enriched in the JEJLP and the JEJEPI (Fig. 4G).

### Distinct features of the infiltration and clinical significance of CD8 resident memory T cells among distinct intestine regions in TCGA-COAD samples

There are increasing evidences for the role of Trm cells in the immune response against cancer in mouse models as well as in clinical patient [12, 31, 37, 40] and evidences indicated that infiltration of Trm cells into the cancer tissue was correlated with a better prognosis and overall survival rates in cancers including breast, lung, ovarian, and cervical [1, 38]. TCR analyses from tissue samples of different diseases or virus infections revealed distinct immune repertoire in circulation and primary tissues which suggests localized responses or specific infiltration of clonally expanded populations [26, 41]. To demonstrate the migration and immune response of the Trm_gut_CD8 from distinct intestine organs in the colorectal cancer sample, we collected the scRNA-seq data [61] of paired normal and cancer samples which included 21 cancer types, 316 patients, and 397810 T cells (Fig. 1A) for the following processing including cancer type filtering and quality control. We perform the mapping and annotation of the query dataset using the above reference dataset [18] (Fig. 5A and Additional file 1: Figure S8A). The Trm_gut_CD8 from DUO, ILE, and JEJEPI showed high levels of cytokines and effector molecules while the Trm_gut_CD8 from the JEJLP showed high levels of inhibitory markers (Fig. 5B). It was implied that the Trm_gut_CD8 from the DUO and the ILE decreased while the Trm_gut_CD8 from the JEJEPI increased in the cancer compared to the normal tissue (Fig. 5C and Additional file 1: Figure S8B). We compared the difference in the gene expression level of the Trm_gut_CD8 between the normal and cancer tissue (Fig. 5D). Several effector molecules including GAMA, TNF, GZMH, and GNLY were up-regulated in the Trm_gut_CD8 of the DUO and the ILE. The analyses of enriched signatures implied that immune response including cytokine production, MHC complex assembly, T cell receptor signaling as well and the apoptotic process were enriched in the Trm_gut_CD8 of the DUO and the ILE while the Trm_gut_CD8 in the JEJEPI were enriched in cell replication, immune response, and negative regulation of apoptotic process (Fig. 5E). In the cancer, the Trm_gut_CD8 showed higher expression levels of cytokines and effector molecules indicating a stronger immune response, and lower levels of resident markers indicating their decreased migration ability (Additional file 1: Figure S8C). Furthermore, the TCGA colorectal cancer data which included 458 patients and 10 distinct cancer stages was collected. We calculated differential genes and enriched signaling pathways in samples with high infiltration of Trm_gut_CD8 cells of distinct organs compared to samples with low infiltration (Additional file 1: Figure S9A, B). It was implied that the great infiltration of Trm_gut_CD8 cells of the DUO and the ILE was associated with T cell activation, proliferation, and the production of related cytokines and chemokines. However, the great infiltration of Trm_gut_CD8 cells of the JEJEPI was associated with several anti-tumor response, extracellular matrix organization, and negative regulation of T cell activation (Additional file 1: Figure S9B). It was implied that samples with greater overall survival showed more infiltration of the Trm_gut_CD8 cells from the DUO and the ILE and less infiltration of the Trm_gut_CD8 cells from the JEJ (Fig. 5F). We also demonstrate the correlation between overall survival and other parameters including primary diagnosis tissues, pathologic stages, and original occurrence tissues (Additional file 1: Figure S9C). Distinct pathological stages were different in the infiltration of the Trm_gut_CD8 (Additional file 1: Figure S9D). We wondered whether the infiltration of Trm_gut_CD8 cells was correlated with the response to immune checkpoint blockade in the colorectal cancer. The scRNA-seq data of colorectal cancer patients receiving anti-PD-1 blockade therapy [25] was collected. We tested the correlation between the infiltration of Trm cells into tumor samples before treatment with response to the immune checkpoint blockade therapy and implied that the infiltration of Trm_gut_CD8 cells of the DUO and the ILE was correlated with the therapy response (Fig. 5G). Above all, it could be suggested that the infiltration of Trm_gut_CD8 cells of the DUO and the ILE could be utilized for the prediction of the overall survival and the immune checkpoint blockade therapy response in the colorectal cancer.

## Discussion

Trm CD8 cell is a specific memory CD8 T cell subset which specifically located in almost all non-lymphoid tissues and is distinct in molecular phenotypes and biological functions compared to the circulating counterparts including effector memory T cells and central memory T cells [28, 29]. Trm CD8 T cells play an important role in robust and immediate immune response including cytotoxicity and cytokine production in tissues upon virus infection or tumorigenesis [2, 13, 16].

Trm CD8 T cells are enriched in the small intestine, located in the LP or EPI, constitute the barrier, and prevent the intestine from pathogen infection. Trm CD8 T cells of distinct intestine regions show heterogeneity and might be derived from distinct precursor cells or migrate from other organs via distinct development pathways which can be assumed that Trm CD8 T cells with distinct phenotypes acquired diverse transcriptomics features and biological functions [23, 33]. Learning the heterogeneity of the intestine Trm CD8 T cells helped us to better understand the immune microenvironment in the intestine which was vital for the intestine's immune response.

However, it was reported that previous research [19, 28, 29] about the memory CD8 T cells was mainly about the circulating counterparts but less focused on the tissue-resident subsets that initiate the primary immune response. Besides, it was not convenient to separate the Trm subset by former technologies and track the development pathway in vivo. Trm CD8 T cells derived from patients of distinct ages always showed acute heterogeneity which might result in batch effector.

In our study, we collected scRNA-seq and scTCR-seq datasets of Trm CD8 T cells from different organs to illustrate their transcriptomic heterogeneity as well as their lineage tracking during their development. We demonstrated a specific Trm CD8 T cell subset named Trm_gut_CD8 that was specifically located in diverse regions of the small intestine. We further demonstrated the transcriptional heterogeneity of the Trm_gut_CD8 and implied that the differentiation of Trm_gut_CD8 in the DUO was mediated by interacting with B cells via several signaling pathways including TNF, CD48, LCK, and MHC-I. Trm_gut_CD8 in the DUO and ILE were related to immune effector processes. WGCNA analyses demonstrated that Trm_gut_CD8 in the DUO and ILE had similar transcriptional features. We utilized the STARTRAC to track the lineage of Trm_gut_CD8 in the small intestine and implied that Trm_gut_CD8 might be derived from the differentiation of the T_CD4/CD8 subset within the same organ or migrated from several organs including SPL and MLN. Trm_gut_CD8 in the JEJEPI and JEJLP showed similar immune repertoire and might be derived from the same precursor T cell subset. The comparative analyses of the immune repertoire of Trm_gut_CD8 from distinct organs also suggested distinct development pathways. To illustrate the dynamic of Trm_gut_CD8 from distinct organs in the tumorigenesis, we demonstrated the infiltration feature of Trm_gut_CD8 of distinct organs. It was also implied that Trm_gut_CD8 of the DUO and the ILE showed effector immune response including inflammation response and cytokine production while Trm_gut_CD8 in the JEJEPI showed cell proliferation in the cancer compared to the normal tissue. The TCGA data also demonstrated that samples with more infiltration of Trm_gut_CD8 of the DUO and the ILE showed greater overall survival. However, there still could be other factors that might contribute to both Trm_gut_CD8 infiltration and improved survival and therefore experimental examinations were needed for the validation. At last, the scRNA-seq data of colorectal samples receiving immune checkpoint blockade implied that patients with greater response showed more infiltration of Trm_gut_CD8 of the DUO and the ILE into the tumor before the treatment which could be utilized as a prognosis marker.

## Method

### Data collection

Published data and TCGA data were collected in this research. scRNA-seq data and scTCR-seq data of the T cell populations among distinct human organs were derived from the Cross-tissue Immune Cell Atlas http://www.tissueimmunecellatlas.org) [11] and the CellTypist database (https://www.celltypist.org/) [57]. The scRNA-seq data of the T cell populations among patient-paired normal and cancer organs was derived from the Gene Expression Omnibus repository (http://www.ncbi.nlm.nih.gov/geo/), and the accession code was GSE156728 [61]. The TCGA data of 548 colorectal cancer samples were collected for the survival analysis. The scRNA-seq data of the colorectal cancer receiving anti-PD-1 blockade therapy was derived from the Gene Expression Omnibus repository (http://www.ncbi.nlm.nih.gov/geo/), and the accession code was GSE205506 [25].

### Processing of scRNA-seq data

The scRNA-seq data was processed by the Scanpy package (version 1.9.5) [53]. The object was created by the function read_h5ad. Barcodes of poor quality were filtered using parameters including min cells 3 and min genes 200. Samples from distinct organs and patients were integrated with Scanpy for further analysis. The total count normalized the data matrix to 10000 reads per cell so that counts became comparable among cells. Highly variable genes were identified. Regress out effects of total counts per cell and the percentage of mitochondrial genes expressed. Scale the data to unit variance. We reduced the dimensionality of the data by running principal component analysis (PCA), which revealed the main axes of variation and denoised the data. The neighborhood graph of cells of the data was computed using the PCA representation following the embedding of the graph in two dimensions using UMAP. The ranking of highly differential genes in each cluster was computed using the t-test.

### Cell type identification

Several marker genes acquired from former researches [11, 17] were utilized for the identification and annotation of T cell populations. The Dotplot function was used to illustrate the expression feature of the T cell. The markers include TCF7, SELL, LEF1, CCR7 (naive markers); CXCR6, NR4A1, CD69, RUNX3, (resident markers); LAG3, TIGIT, PDCD1, HAVCR2, CTLA4, (inhibitory and exhausted markers); IL2, GZMA, GNLY, PRF1, GZMB, GZMK, IFNG, NKG7, TNFSF10, CST7, PRF1, (cytokines and effector molecules); CD28, TNFRSF14, ICOS, TNFRSF9, CD226, TNFRSF25, (co-stimulatory molecules); EOMES, HOPX, TBX21, ZEB2, ZNF683, HIF1A, ID2, TOX, (transcription factors); IL2RA, FOXP3, IKZF2, (Treg markers); CD4, CD3G, CD3D, CD3E, CD8A, CD8B, KLRF1, KLRD1 (pan-T cell markers).

### Subpopulation analysis of T cells

For further analyses of the Trm_gut_CD8 subset, we selected Trm_gut_CD8 among samples and organs and combined them into an integrated object [53]. The following clustering, embedding, and differential genes calculations were as same as the steps above.

### Identification of Trm_gut_CD8 signature and PPI network analysis

To identify specific signatures of the Trm_gut_CD8 subset, we calculated differential genes using the findmarkers function [43] and filtered the result with parameters as follows: avg_log2FC > 2 and p_val_adj < 0.001. Then the filtered gene list was submitted to STRING [45] to obtain the gene module which was annotated as the Trm_gut_CD8 gene module. The selected differential genes were used to construct the PPI network in the STRING (http://cn.string-db.org/). Genes related to specific signatures were selected and colored.

### Gene ontology (GO) analysis

The GO analyses of several interested clusters were calculated using packages including clusterProfiler [55], GSEABase, GO.db, org.Hs.eg.db, and AnnotationHub. We used the enrichGO function to calculate the significance of signaling pathways with parameters as follows: OrgDb is org.Hs.eg.db, pAdjustMethod is BH, and pvalueCutoff is 0.05. Finally, significant GO terms were selected and visualized with ggplot2.

### Receptor & ligand analysis

We analyzed the differences in receptor and ligand communication between Trm_gut_CD8 and other cells with R packages including CellChat [21], NMF, and ggalluvial. The cellchat object was constructed by importing the gene expression matrix and the annotation of sub-structures. The human receptor & ligand database was imported as the reference. The signal pathway-related genes were extracted from the gene expression matrix. Receptors and ligands that were highly expressed in each cluster were searched which were projected onto the protein–protein interaction network. The interaction between the overexpressed ligand and receptor is identified.

### WGCNA analysis

We used the hdWGCNA [34] package to perform the weighted gene co-expression network analysis in the scRNA-seq dataset which identified robust modules of interconnected genes. We will set up the Seurat object using the SetupForWGCNA function, construct metacells in each cell subset using the MetacellsByGroups function, set up the expression matrix using the SetDatExpr function, select soft-power threshold using the TestSoftPowers function, and do the co-expression network analysis to construct the co-expression network and visualize different co-expression modules of each Trm_gut_CD8 subset.

### T cell lineage analysis

We performed the T-cell lineage analysis using STARTRAC [59] (Single T-cell Analysis by Rna-seq and Tcr TRACking) to illustrate the expansion, migration, and transition with the scRNA-seq data and the scTCR-seq data. The STARTRAC-expa index indicated the clonal expansion of T cells. The STARTRAC-migr index indicated the cross-tissue migration of T cells. The STARTRAC-tran index indicated the state transition of T cells. The STARTRAC-gini index indicated the similarity of T cell clonotypes within the same organ. We further calculated the composition of T cells of distinct clone sizes, and compared the overlap pattern of T cell subsets from distinct organs as supporting materials. Using trajectory-related R packages including Monocle [7] and Biobase, we constructed the pseudo-time trajectory of distinct T cell populations within the same organ to illustrate the difference pathway of Trm_gut_CD8.

### Immune repertoire analysis

Since distinct Trm_gut_CD8 differentiated in distinct pathways and were assumed to have distinct features in the immune repertoire, we can compare the differences in distinct Trm_gut_CD8 subsets. We compared differences in VJ gene usage, CDR3 amino acid sequence length, clonotype size dynamic, the composition of amino acids with specific physicochemical features in specific positions [24], and their overall distribution.

### Deconvolution pipeline and survival analysis

To infer the cellular composition of T cells in bulk RNA-seq data downloaded from the TCGA database, the bulk RNA-seq data was deconvoluted by BayesPrism [9]. The scRNA-seq data of all T cell subsets was used to construct the prism object after quality control and outlier genes filter. After running the BayesPrism pipeline, the predicted composition of cell types in distinct TCGA samples was acquired. We calculated the differential genes of samples with high infiltration or low infiltration of Trm_gut_CD8 cells of distinct organs. We also calculated the differential genes of each T cell subset and module scores for further grouping in the survival analysis. We illustrated the correlation between the infiltration of distinct Trm_gut_CD8 and the overall survival rate in colorectal cancer samples.

### Statistical analysis

All statistical analyses were performed by R with Student’s t-test and log-rank test in which results were statistically significant if the p < 0.05.

### Supplementary Information

**Additional file 1: Figure S1.** Preprocessing of the scRNA-seq dataset. A Box plots indicating the gene count, UMI count, and mitochondrial gene percent in each donor. B Box plots indicating the gene count, UMI count, and mitochondrial gene percent in each T cell subset. C Box plots indicating the gene count, UMI count, and mitochondrial gene percent in each organ. D Point plot indicating the dispersions and mean expression of highly variable genes. E The box plot indicates genes with the highest fraction of counts in each single cell. F UMAP plot showing single cells collected from the CellTypist database, labeled by organs (left), library construction methods (middle), and donors (right). **Figure S2.** The distribution of T cell subsets in distinct organs. A Bar plots indicating the overall barcode count of distinct donors (left) and organs (middle) and the proportion of each cell subsets among organs (right). B Box plots indicating the proportion of T cell subsets in distinct organs. C Heatmaps indicating the clustering of T cell subsets based on their distribution in distinct organs. **Figure S3.** The transcriptomic feature of distinct organs and T cell subsets. A The dot plot indicates the expression of several marker genes in distinct organs. B The point plot indicating differential genes of T cell subsets. Genes with log2Foldchange greater than 2 and p value adjust less than 0.001 were annotated as differential genes. C Heatmaps of the overlapping of differential genes among T cell subsets. Cells with a proportion of differential genes greater than 0.7 are labeled as “ +  +  + ”. Cells with a proportion of differential genes greater than 0.5 are labeled as “ +  + ”. Cells with a proportion of differential genes greater than 0.3 are labeled as “ + ”. D The point plot indicating differential genes of the Trm_gut_CD8. Genes with abs(log2Foldchange) greater than 2 and p value adjust less than 0.001 were annotated as differential genes. E The bar plot indicating enriched pathways of Trm_gut_CD8. **Figure S4.** The transcriptomic feature of the Trm_gut_CD8 subset. A The dot plot indicates the top5 differential genes of the Trm_gut_CD8 among distinct intestine organs. B Heatmaps of the overlapping of differential genes Trm_gut_CD8 among distinct intestine organs. C The bar plot indicating enriched pathways of PPI gene modules of Trm_gut_CD8 among distinct intestine organs. **Figure S5.** The receptor & ligand analysis of cell subsets in the DUO organ. A Circle plots indicating interaction numbers among cell subsets in the DUO. B Circle plots indicating interaction strength among cell subsets in the DUO. C Circle plots of the CD48 signaling pathway network and specific receptor & ligand pairing among cell subsets. D Circle plots of the TNF signaling pathway network and specific receptor & ligand pairing among cell subsets. E Circle plots of the LCK signaling pathway network and specific receptor & ligand pairing among cell subsets. F Left, circle plots of the MHC-I signaling pathway network among cell subsets. Right, the bar plot indicates the contribution of distinct receptor & ligand pairing patterns. Contribution greater than 0.01 was labeled as “detected”. **Figure S6.** The WGCNA analysis of the Trm_gut_CD8 from distinct intestine organs. A The summary figure of the soft-power threshold selection. B The corrplot indicating the correlation between each gene module based on their hMEs, MEs, or hub gene scores. C Top 25 hub genes in each module ranked by kME using the PlotKMEs function. D Bar plots indicating the gene ontology analyses and KEGG analyses of hub genes of each gene module. **Figure S7.** T cell subsets lineage tracking and the clonotypes comparison of distinct T cell subsets. A Box plots indicating index including STARTRAC-expa, STARTRAC-migr, STARTRAC-tran, STARTRAC-gini in distinct T cell subsets. B Bar plots indicating the clonotype expansion degree of T cell subsets in four intestine organs. C Heatmaps indicating the overlapping of clonotypes of distinct T cell subsets within specific organs. D Heatmaps indicating the overlapping of clonotypes of Trm_gut_CD8 among distinct organs. E Heatmaps indicating the overlapping of clonotypes of distinct T cell subsets among distinct organs. **Figure S8.** The distribution and transcriptomic feature of the Trm_gut_CD8 in paired tumor and normal samples. A tSNE plots indicating the T cell subsets in the query dataset split by tumor and normal samples. B Violin plots indicating predicted percent of Trm_gut_CD8 derived from distinct organs between normal and tumor tissues. C Dot plots indicating the expression levels of marker genes in Trm_gut_CD8 derived from distinct organs between normal and tumor tissues. **Figure S9.** The infiltration of Trm_gut_CD8 cells in the colorectal cancer benefits the overall survival and the response to the immune checkpoint blockade therapy. A Scatter plots indicating differential genes of TCGA colorectal samples with high infiltration of Trm_gut_CD8 cells in distinct organs. B The bar plot indicating the enriched signaling pathways of up-regulated genes in the panel A. C Overall survival curves of colorectal cancer patients stratified by

## Data Availability

All relevant data are within the paper and its Supporting Information files.
