# Supplementary material for: Decoding the transcriptional heterogeneity, differentiation lineage, clinical significance in tissue-resident memory CD8 T cell of the small intestine by single-cell analysis
Source: J Transl Med. 2024 Feb 25;22:203. doi: 10.1186/s12967-024-04978-2 (PMC10895748; doi:10.1186/s12967-024-04978-2)
Supplement: Supplementary file 1 — Additional file 1: Figure S1. Preprocessing of the scRNA-seq dataset. A Box plots indicating the gene count, UMI count, and mitochondrial gene percent in each donor. B Box plots indicating the gene count, UMI count, and mitochondrial gene percent in each T cell subset. C Box plots indicating the gene count, UMI count, and mitochondrial gene percent in each organ. D Point plot indicating the dispersions and mean expression of highly variable genes. E The box plot indicates genes with the highest fraction of counts in each single cell. F UMAP plot showing single cells collected from the CellTypist database, labeled by organs (left), library construction methods (middle), and donors (right). Figure S2. The distribution of T cell subsets in distinct organs. A Bar plots indicating the overall barcode count of distinct donors (left) and organs (middle) and the proportion of each cell subsets among organs (right). B Box plots indicating the proportion of T cell subsets in distinct organs. C Heatmaps indicating the clustering of T cell subsets based on their distribution in distinct organs. Figure S3. The transcriptomic feature of distinct organs and T cell subsets. A The dot plot indicates the expression of several marker genes in distinct organs. B The point plot indicating differential genes of T cell subsets. Genes with log2Foldchange greater than 2 and p value adjust less than 0.001 were annotated as differential genes. C Heatmaps of the overlapping of differential genes among T cell subsets. Cells with a proportion of differential genes greater than 0.7 are labeled as “ + + + ”. Cells with a proportion of differential genes greater than 0.5 are labeled as “ + + ”. Cells with a proportion of differential genes greater than 0.3 are labeled as “ + ”. D The point plot indicating differential genes of the Trm_gut_CD8. Genes with abs(log2Foldchange) greater than 2 and p value adjust less than 0.001 were annotated as differential genes. E The bar plot indicating enriched pat [file 12967_2024_4978_MOESM1_ESM.docx]

**Fig s1**

**Fig s1. Preprocessing of the scRNA-seq dataset.**

1. Box plots indicating the gene count, UMI count, and mitochondrial gene percent in each donor.
2. Box plots indicating the gene count, UMI count, and mitochondrial gene percent in each T cell subset.
3. Box plots indicating the gene count, UMI count, and mitochondrial gene percent in each organ.
4. Point plot indicating the dispersions and mean expression of highly variable genes.
5. The box plot indicates genes with the highest fraction of counts in each single cell.
6. UMAP plot showing single cells collected from the CellTypist database, labeled by organs (left), library construction methods (middle), and donors (right).

**Fig s2**

**Fig s2. The distribution of T cell subsets in distinct organs.**

1. Bar plots indicating the overall barcode count of distinct donors (left) and organs (middle) and the proportion of each cell subsets among organs (right).
2. Box plots indicating the proportion of T cell subsets in distinct organs.
3. Heatmaps indicating the clustering of T cell subsets based on their distribution in distinct organs.

**Fig s3**

**Fig s3. The transcriptomic feature of distinct organs and T cell subsets.**

1. The dot plot indicates the expression of several marker genes in distinct organs.
2. The point plot indicating differential genes of T cell subsets. Genes with log2Foldchange greater than 2 and p value adjust less than 0.001 were annotated as differential genes.
3. Heatmaps of the overlapping of differential genes among T cell subsets. Cells with a proportion of differential genes greater than 0.7 are labeled as “+++”. Cells with a proportion of differential genes greater than 0.5 are labeled as “++”. Cells with a proportion of differential genes greater than 0.3 are labeled as “+”.
4. The point plot indicating differential genes of the Trm_gut_CD8. Genes with abs(log2Foldchange) greater than 2 and p value adjust less than 0.001 were annotated as differential genes.

**Fig s4**

**Fig s4. The transcriptomic feature of the Trm_gut_CD8 subset.**

1. The dot plot indicates the top5 differential genes of the Trm_gut_CD8 among distinct intestine organs.
2. Heatmaps of the overlapping of differential genes Trm_gut_CD8 among distinct intestine organs.
3. The bar plot indicating enriched pathways of PPI gene modules of Trm_gut_CD8 among distinct intestine organs.

**Fig s5**

**Fig s5. The receptor & ligand analysis of cell subsets in the DUO organ.**

1. Circle plots indicating interaction numbers among cell subsets in the DUO.
2. Circle plots indicating interaction strength among cell subsets in the DUO.
3. Circle plots of the CD48 signaling pathway network and specific receptor & ligand pairing among cell subsets.
4. Circle plots of the TNF signaling pathway network and specific receptor & ligand pairing among cell subsets.
5. Circle plots of the LCK signaling pathway network and specific receptor & ligand pairing among cell subsets.
6. Left, circle plots of the MHC-I signaling pathway network among cell subsets. Right, the bar plot indicates the contribution of distinct receptor & ligand pairing patterns. Contribution greater than 0.01 was labeled as “detected”.

**Fig s6**

**Fig s6. The WGCNA analysis of the Trm_gut_CD8 from distinct intestine organs.**

1. The summary figure of the soft-power threshold selection.
2. The corrplot indicating the correlation between each gene module based on their hMEs, MEs, or hub gene scores.
3. Top 25 hub genes in each module ranked by kME using the PlotKMEs function.
4. Bar plots indicating the gene ontology analyses and KEGG analyses of hub genes of each gene module.

**Fig s7**

**Fig s7. T cell subsets lineage tracking and the clonotypes comparison of distinct T cell subsets.**

1. Box plots indicating index including STARTRAC-expa, STARTRAC-migr, STARTRAC-tran, STARTRAC-gini in distinct T cell subsets.
2. Bar plots indicating the clonotype expansion degree of T cell subsets in four intestine organs.
3. Heatmaps indicating the overlapping of clonotypes of distinct T cell subsets within specific organs.
4. Heatmaps indicating the overlapping of clonotypes of Trm_gut_CD8 among distinct organs.
5. Heatmaps indicating the overlapping of clonotypes of distinct T cell subsets among distinct organs.

**Fig s8**

**Fig s8. The distribution and transcriptomic feature of the Trm_gut_CD8 in paired tumor and normal samples.**

1. tSNE plots indicating the T cell subsets in the query dataset split by tumor and normal samples.
2. Violin plots indicating predicted percent of Trm_gut_CD8 derived from distinct organs between normal and tumor tissues.
3. Dot plots indicating the expression levels of marker genes in Trm_gut_CD8 derived from distinct organs between normal and tumor tissues.

**Fig s9**

**Fig s9. The infiltration of Trm_gut_CD8 cells in the colorectal cancer benefits the overall survival and the response to the immune checkpoint blockade therapy.**

1. Scatter plots indicating differential genes of TCGA colorectal samples with high infiltration of Trm_gut_CD8 cells in distinct organs.
2. The bar plot indicating the enriched signaling pathways of up-regulated genes in the panel A.
3. Overall survival curves of colorectal cancer patients stratified by primary diagnosis tissue, pathologic stage, and original tissues.
4. Box plots indicating the infiltration scores of Trm_gut_CD8 in distinct pathological stages.
